# Sponsorship and Career Advancement for Asian Medical Faculty

**DOI:** 10.1001/jamanetworkopen.2025.53241

**Published:** 2026-01-09

**Authors:** Dale Sebastian, Karina Gonzalez Herrera, Mohini Ranganathan, Angeli Landeros-Weisenberger, Darin Latimore

**Affiliations:** 1 Yale School of Medicine, Yale University, New Haven, Connecticut; 2 Department of Psychiatry, Yale School of Medicine, Yale University, New Haven, Connecticut; 3 Yale Child Study Center, Yale School of Medicine, Yale University, New Haven, Connecticut; 4 Department of Internal Medicine, Yale School of Medicine, Yale University, New Haven, Connecticut

## Abstract

**Importance:**

Asian faculty members remain underrepresented in leadership positions within academic medicine, despite comprising a substantial proportion of the workforce. The structural and cultural factors associated with the slower advancement remain underexplored in empirical literature and institutional policy.

**Objectives:**

To examine the barriers to career advancement among Asian faculty members, assess perceptions and understanding of sponsorship, and identify institutional strategies to promote broader access to sponsorship opportunities and leadership development.

**Design, Setting, and Participants:**

This qualitative study employed 5 focus groups conducted between January 18 and July 17, 2024, supplemented by real-time polling before and after an educational intervention. Thematic analysis of transcripts was used to identify common barriers and recommendations, with poll data providing a descriptive context. In a single, large private nonprofit and research intensive academic medical center in the northeastern United States, self-identified Asian or Asian American midlevel faculty members at the associate professor level participated. Participants were recruited via institutional office outreach, and responses from all recruited participants were included in the study. Data were analyzed from July 25 to September 24, 2024.

**Exposure:**

An educational session distinguishing sponsorship from mentorship, followed by a facilitated group discussion and anonymous survey participation using the Poll Everywhere platform.

**Main Outcomes and Measures:**

Primary outcomes included changes in participants’ self-reported understanding of sponsorship and identification of perceived personal, institutional, and departmental barriers to career advancement. Thematic categories were derived from focus group transcripts and corroborated with quantitative poll responses.

**Results:**

Of the 32 participants who completed the survey, 18 (56.3%) initially believed that they had a sponsor; after the intervention, this percentage decreased to 37.5% (12 of 32) once sponsorship was more clearly defined. The participation rate was 93.7% (30 of 32) among those who joined the focus groups. Reported barriers included cultural norms discouraging self-promotion, limited access to decision-makers, intersectional identity challenges, and perceptions of institutional opacity and favoritism. Faculty emphasized the value of transparent nomination processes, intentional sponsor training, and relationship-centered sponsorship models.

**Conclusions and Relevance:**

In this qualitative study of midlevel Asian faculty members, participants faced persistent, multifactorial barriers to leadership in academic medicine, amplified by cultural values and institutional norms. Institutions must formalize sponsorship programs, ensure transparent advancement pathways, and foster culturally informed leadership development. Doing so may meaningfully advance deserving Asian faculty members and diversify academic medicine’s leadership pipeline.

## Key Points

**Question:**

What barriers do midlevel Asian faculty members face in achieving career advancement in academic medicine, and how is sponsorship perceived as a potential solution?

**Findings:**

In this qualitative study at a large academic medical center, 32 self-identified Asian or Asian American midlevel faculty members reported barriers, including cultural norms discouraging self-promotion, limited access to decision-makers, and unclear pathways to sponsorship. Although sponsorship was viewed as essential for advancement, fewer participants believed they had a sponsor after receiving a formal definition.

**Meaning:**

These findings suggest that academic medical institutions may need intentional, relationship-centered sponsorship initiatives to address unique advancement challenges experienced by Asian faculty members and illuminate opaque leadership pathways.

## Teaser

This qualitative study examines the barriers to career advancement among Asian faculty members, assesses perceptions and understanding of sponsorship, and identifies institutional strategies to promote broader access to sponsorship opportunities and leadership development.

## Introduction

Asian faculty members represent a significant portion of the academic-medicine workforce yet remain underrepresented in leadership roles. According to 2022 Association of American Medical Colleges data, although Asian individuals represent 20.5% of US medical school faculty, only 15.4% attain full professorship, and just 10.1% hold department-chair positions.^1,2^ This persistent gap—despite high entry into academic medicine—reflects an often-invisible barrier to advancement commonly described as the “bamboo ceiling.”^3,4^

The bamboo ceiling encompasses cultural, interpersonal, and structural factors that restrict upward mobility for Asian professionals, including implicit bias, limited access to mentorship and sponsorship, and cultural norms that discourage self-advocacy.^4,5^ These challenges are compounded by the model-minority myth, which falsely assumes that Asian faculty members do not experience discrimination and, as a result, do not require support within diversity, equity, and inclusion initiatives.^6^

Although Asian faculty members are not formally designated as underrepresented in medicine, emerging evidence has demonstrated disparities in promotion, recognition, and leadership opportunity similar to those experienced by other marginalized groups.^7^ Intersectional identities across race, sex, nationality, immigration status, and educational background may further exacerbate these disparities, particularly for midcareer faculty members.^8,9^

These perceptions contribute to a false narrative that racial and ethnic equity for Asian professionals has already been achieved, perpetuating invisibility, limiting institutional support, and reinforcing assumptions that they do not require advocacy or advancement opportunities.^6,10^ Additionally, the “perpetual-foreigner” bias continues to undermine Asian faculty members’ sense of belonging and recognition within academic institutions.^11,12^

There is growing recognition that, although mentorship is essential for early-career support, it alone is insufficient for propelling faculty—particularly women and historically excluded groups—into positions of leadership and influence.^13,14^ Sponsorship, in contrast, involves active advocacy, including nominating individuals for leadership roles, connecting them to high-impact networks, and creating tangible career opportunities.^15,16^

Despite its growing importance, little is known about how Asian faculty members understand or access sponsorship and what institutional structures support—or hinder—its equitable distribution.^17^ In this qualitative study, we aimed to (1) assess perceptions and understanding of sponsorship, (2) examine the perceived career-advancement barriers experienced by midlevel Asian faculty members, and (3) identify institution-level strategies for advancing equity based on faculty input.

## Methods

### Participants, Sampling, and Recruitment

This qualitative study followed the Consolidated Criteria for Reporting Qualitative Research (COREQ) reporting guideline. In this qualitative study using purposive sampling, we recruited midlevel Asian faculty members at a large academic medical center whose ranks and lived experiences positioned them to reflect on barriers to career advancement and perceptions of sponsorship. Recruitment was facilitated through the Yale School of Medicine Office of Collaborative Excellence (formerly Office of Diversity, Equity and Inclusion) and the Yale Asian Faculty Association. Approximately 164 faculty members were invited via email to participate in 1 of 5 scheduled focus groups conducted between January 18 and July 17, 2024; 33 participated, of whom 30 completed the study with no exclusions or triage applied beyond inclusion criteria. Inclusion criteria included participant self-identification as Asian or Asian American and current appointment at the associate professor level or higher. All participants provided informed consent prior to participation, including permission to publish anonymous quotations. Race and ethnicity were self-reported. No incentives were offered. Focus groups continued until thematic saturation was reached (ie, when no new themes emerged across sessions). The study received an institutional review board exemption from the Yale University Human Research Protection Program as a minimal-risk survey and disclosure of responses would not reasonably place participants at risk of harm.

### Data Collection

We developed a semistructured focus group guide to examine participants’ perceptions of sponsorship, challenges in leadership advancement, and recommendations for institutional improvement. The discussion framework included educational slides defining sponsorship, contrasting it with mentorship, and illustrating examples of sponsorship activities (eg, nominations for awards and introductions to networks). Sessions were cofacilitated by trained physicians (D.L. and D.S.) familiar with qualitative interviewing.

Discussions lasted approximately 60 minutes and were transcribed in real time by trained assistants (K.G.H., M.R., and A.L.-W.), with cross-verification for accuracy. Demographic data, including academic track and sex, were self-reported by the participants and collected anonymously using the Poll Everywhere platform.^18^ Participants responded to initial questions regarding their understanding of sponsorship before and after the educational slides.

### Statistical Analysis

Data analysis was conducted from July 25 to September 24, 2024. Investigators independently coded all transcripts using an inductive approach to identify recurring concepts and themes associated with career barriers and sponsorship experiences. A codebook was developed iteratively through consensus meetings. Representative quotes were selected to illustrate major themes. The team included faculty members with expertise in medical education, health equity, and faculty development. We used thematic analysis to generate higher-order themes and domains and used NVivo software, version 12 (QSR International) to manage and organize data. Poll data were descriptively analyzed and used to contextualize the qualitative themes.

## Results

### Participant Characteristics

Thirty-two midlevel Asian faculty members at the associate professor rank participated in the study, with 32 completing only the survey and 30 completing both the focus group and survey. Of the 30 participants, 17 (56.7%) self-identified as male, and 13 (43.3%) as female. All participants held the academic rank of associate professor or higher and were employed at a single academic medical center in the northeastern United States. Of the group, 43.3% (13 of 30) held tenure-track positions (research focused with tenure eligibility), 33.3% (10 of 30) identified as clinician-scientists (balanced clinical research), 10.0% (3 of 30) identified as academic clinicians (primarily clinical), and 10.0% (3 of 30) identified as clinician educator-scholars (education focused). Although participants discussed their specific Asian ethnic origins during the focus groups, this information was not formally collected in the demographic survey.

### Understanding Sponsorship

Prior to receiving a formal definition, 56.3% (18 of 32) of participants believed they had a sponsor. However, after learning that sponsorship entails active advocacy—such as nominations and access to high-impact networks—that percentage decreased to 37.5% (12 of 32). Participants often struggled to differentiate sponsorship from mentorship.

One faculty member reflected, “Someone encouraged me to apply for opportunities—but was that mentorship or sponsorship? It’s hard to tell.” Others expressed skepticism regarding the motivations behind sponsorship, noting uncertainty about whether such support was altruistic or transactional. As 1 participant questioned, “Why would a senior person do this for me? They are very busy—what’s in it for them?”

### Individual-Level Barriers to Career Advancement

Participants commonly cited cultural values that discourage self-advocacy, particularly among those from collectivist cultures or immigrant backgrounds. These norms often conflicted with the expectations of academic medicine. These cultural frames created a reluctance to seek sponsorship, even when participants had strong records of accomplishment.

Psychological factors, including imposter syndrome, fear of rejection, and discomfort with assertiveness, further contributed to hesitancy. Social disconnection was also a recurring theme, with several participants describing difficulties navigating common informal networking environments.

Structural constraints, such as heavy clinical workloads, lack of protected time, and caregiving responsibilities, further limited access to leadership pathways. Others noted that being seen as reliable and productive often came at the cost of decreased visibility for leadership consideration. Table 1 provides illustrative quotations for each theme.

**Table 1.** Thematic Summary of Personal Barriers to Career Advancement

Theme	Subthemes	Illustrative quotations
Cultural differences	Cultural norms discouraging self-promotion; immigration-related values	“Self-promotion wasn’t something we were taught—it often felt discouraged and uncomfortable.”
Emotional and psychological	Imposter syndrome; fear of rejection; introversion; prior marginalization; social disconnection	“Speaking up was discouraged growing up, so confidence and fear of being wrong remain challenges.” “Sometimes I’m at a dinner interview and the conversation is all about wine and golf. I have no idea what to say.”
Deference to hierarchy and authority	Respect for elders; difficulty navigating peer relationships with senior faculty	“I was raised to defer to elders; treating senior colleagues as peers still feels unnatural.”
Work and time constraints	Heavy clinical workloads, absence of protected time, and prioritization of patient care	“We’re hired to do the hard work—there’s little time left for visibility or networking.”
Parental and familial commitments	Caregiving responsibilities; limited access to informal networking	“Evening events and networking happen while I’m at home managing bedtime.”

### Intersectionality and Compounding Identity-Based Barriers

Intersectionality emerged as a critical theme throughout participants’ narratives, emphasizing how multiple facets of identity—such as sex, nationality, educational background, and religious affiliation—intersect to shape access to opportunities for career advancement. Table 2 provides illustrative quotations for each theme.

**Table 2.** Thematic Summary of Intersectionality and Compounding Identity Based Barriers

Theme	Subthemes	Illustrative quotations
Language fluency	Accent bias affecting perceived competence	“Certain accents are perceived as more prestigious, while others may lead to assumptions about lower education or competence.” “The moment you walk in, you’re put in a box. If they hear an accent… it can lock you out.”
Educational pedigree	Skepticism toward foreign graduates from non-Western institutions	“I’m from a small institution back home…. It feels like they don’t consider you as qualified.” “I felt I had to earn extra degrees just to be seen as equal to US-trained peers.”
Sex	Social expectations and exclusion from informal events among women	“As a woman, especially from a minority background, self-promotion feels uncomfortable and often discouraged.”
Skin tone	Biases related to nationality and appearance	“Opportunities often favor the ‘ideal’ candidate—a White, Ivy League–trained physician—while IMGs still face subtle bias.”
Religious identity	Discomfort in shared spaces	“Religious identity contributes to discomfort.”

Abbreviation: IMG, international medical graduate.

### Departmental and Institutional Challenges to Career Advancement

Many faculty members perceived entrenched institutional biases tied to race and ethnicity, accent, educational pedigree, and perceived “fit.” Such biases manifested in assumptions about competence and diminished access to leadership pipelines. Participants also described being saddled with invisible labor without equivalent recognition. Many participants underscored their limited exposure to senior leadership or key decision-makers. Opportunities for career advancement were frequently described as emerging from informal networks or exclusive social ties, rather than from formal, transparent processes. At the time of the study, the institution lacked formal structured sponsorship programs, although mentorship opportunities existed through departmental initiatives. Participants consistently reported a perception that a select group of faculty members were repeatedly chosen for awards, committees, and high-visibility roles. Table 3 provides illustrative quotations for each theme.

**Table 3.** Thematic Summary of Departmental and Institutional Barriers to Career Advancement

Theme	Subthemes	Illustrative quotation
Invisibility	Implicit bias; departmental favoritism; lack of diversity in leadership; certain identities repeatedly nominated	“Despite my qualifications, I often feel invisible in departmental decisions—opportunities go to a familiar few who resemble current leaders.”
Not part of the inner circle	Limited interaction with potential sponsors; minimal exposure to leadership; exclusions from informal networks and social groups	“Access to advancement depends on being in the inner circle—those outside it rarely hear about opportunities.”
Transparency and accountability	Unclear nomination processes; concentrated power; lack of oversight	“Leadership selection feels opaque, with little accountability or clear criteria for advancement.”
Underrepresentation in leadership	Few Asian faculty members in key decision-making positions	“If leadership doesn’t reflect the diversity it claims to value, what message does that send?”

### Desired Sponsorship Activities and Recommendations

In response to specific prompts about preferred sponsorship activities, participants highlighted 3 high-impact forms: (1) award nominations to enhance visibility, (2) nominations for leadership roles, and (3) collaborative research on high-impact projects with senior faculty. See Table 4 for representative quotations.

**Table 4.** Faculty Recommendations and Illustrative Comments on Institutional Strategies to Promote Equitable Sponsorship

Solutions	Description	Illustrative quotation
Recognition and awards	Nominate Asian faculty members for leadership roles, awards, and national presentations.	“Visibility leads to opportunity. A single nomination—for an award or a talk—can change a career trajectory.”
Promotion and advancement	Clarify pathways to promotion; create nonclinical leadership roles with titles.	“You’re doing the work, but without a title or recognition, it’s like you’re invisible.”
Networking and visibility	Create informal settings and forums for faculty to connect across ranks.	“Opportunities come from being seen—when senior faculty introduce us or nominate us, it signals that we belong.”
Transparency and accessibility	Centralized portal for easy accessibility, ensure transparent access to grant roles, project collaborations, and DEI leadership.	“If no one knows how nominations happen, it feels arbitrary. Transparency builds trust.”
Leadership development and training	Assign formal sponsors; provide leadership training; integrate sponsorship into chair evaluations.	“Sponsorship needs to be taught, modeled, and tied to chair evaluations.”
Accountability	Implement metrics and nomination committees; track sponsorship activity across departments.	“Sponsorship must be intentional and measured—not just based on who leaders ‘feel like’ supporting.”

Abbreviation: DEI, diversity, equity, and inclusion.

### Attributes of an Effective Sponsor

Participants described an effective sponsor as both a respected leader and a relational ally—someone who combines institutional influence with personal integrity. Professional credibility and visibility were seen as prerequisites: “Sponsors need to be well respected, with a significant network and a record of helping others advance.” Yet beyond reputation, participants emphasized human qualities that foster psychological safety and trust. Empathy, kindness, and genuine interest in the individual’s growth were repeatedly highlighted: “A good sponsor is kind, caring, and genuinely wants to know you as a person and your aspirations.” Many noted that integrity and fairness were essential to building confidence in the sponsorship relationship—“Transparency and commitment to the faculty member’s growth matter more than titles,” 1 faculty member shared. Participants also underscored the importance of open-mindedness and inclusion, valuing sponsors who “take risks,” are “nonjudgmental,” and “give you the benefit of the doubt.”

## Discussion

This study identified limited awareness of sponsorship among midcareer Asian faculty members, highlighting the need for targeted education distinguishing it from mentorship and for institutional strategies that support equitable advancement and leadership development. Our findings reinforce previous studies indicating that Asian faculty members, although not underrepresented in medicine overall but in leadership, experience barriers including implicit bias and cultural barriers that overlap with those faced by other groups (eg, women faculty).^10,19^ This contribution highlights how intersectionality and cultural dimensions further diminish visibility, self-advocacy, and recognition. These results align with prior work describing the cumulative impact of racial microaggressions, the perpetual foreigner bias, and exclusion from informal networks, which together perpetuate systemic invisibility and limit access to sponsorship and leadership opportunities.^10,20,21,22,23^

### Cultural Dimensions and Psychological Barriers

In many Asian cultures, deference to authority figures and an emphasis on modest self-presentation are deeply rooted, shaping how individuals negotiate professional relationships.^4,24^ For instance, an ingrained respect for senior colleagues and the norm of avoiding overt self-promotion can collide with Western academic norms that reward assertiveness and visibility.^25,26^ Consequently, Asian faculty members may be less inclined to seek high risk with the potential for high reward opportunities, thus exhibiting greater risk aversion in career decisions.

Imposter syndrome—commonly characterized by persistent self-doubt and an internalized fear of being exposed as a “fraud”—is frequently reported among racial and ethnic minority groups, including Asian faculty members in academic medicine.^25,26,27^ Cultural expectations emphasizing humility and collective harmony can exacerbate self-critical tendencies, leading Asian faculty members to underestimate their accomplishments or question their legitimacy in competitive environments.^24,26,27^ Faculty members may also attribute professional successes to luck or external factors, rather than acknowledging their own expertise.^24^

Fear of rejection emerges as a parallel concern. The reluctance to seek sponsorship for leadership roles may reflect apprehension about being negatively judged by superiors, colleagues, or gatekeeping committees.^16,26,27^ In cultures where “losing face” carries significant social consequences, the prospect of a failed application or visible professional setback can deter individuals from pursuing high-stakes opportunities.^28^ The tension between communal cultural values and Western leadership norms—where self-assertion is often regarded as a marker of competence—may lead some Asian faculty members to avoid networking events, competitive leadership programs, or self-nomination opportunities, thereby inadvertently limiting their access to advancement pathways.^25^

### Complexity of Intersectional Identities

Intersectionality—the overlapping of multiple social identities such as race, sex, nationality, and educational background—creates a complex landscape for Asian faculty members navigating academic medicine.^8,29,30^ Although certain cultural or racial and ethnic factors may appear dominant, participants in this study emphasized that each individual carries a unique constellation of intersecting identities, making it difficult to pinpoint a single, universal barrier. For instance, an Asian woman from a small, non-Western medical institution may experience compounded challenges related to both sex-based discrimination and perceived educational pedigree.^8,29,30^ Another faculty member might additionally contend with accent bias or religious differences, layering yet another barrier on top of racial and ethnic stereotyping.^25^ These multifaceted identities can produce heightened vulnerability, where standard interventions—such as basic mentorship programs—fail to address the nuanced needs of each individual.^8,29,30^

### Stereotypes, Informal Networks, and Role Constraints

Asian faculty members in academic medicine frequently contend with the model minority stereotype, which portrays them as highly competent in clinical or research roles but ill suited for leadership or administrative positions—a bias that reinforces the bamboo ceiling.^10,25,26^ This perception often results in a disproportionate number of Asian faculty members being assigned to clinical or laboratory duties that do not directly contribute to promotion, unlike peers granted protected time for scholarly activities. This is further compounded by the “minority tax,” in which underacknowledged groups take on additional diversity-related responsibilities that detract from academic advancement.^25,30^ As institutional diversity initiatives increasingly highlight other underrepresented in medicine groups, many Asian faculty members report feeling professionally sidelined, contributing to stagnation and burnout.

In many academic settings, informal networks or inner circles play a critical role in the distribution of leadership opportunities, awards, and promotions.^16,20,29^ Access to these circles often depends on shared cultural interests or leisure activities, which may feel unfamiliar or inaccessible to Asian faculty members, particularly those without prior exposure to such norms.^24,26^ Traditional Asian cultural values that emphasize deference to authority can further inhibit the informal self-promotion or bold introductions often necessary to build rapport with senior colleagues.^24,26^ As a result, Asian faculty members may remain invisible in key decision-making forums and excluded from sponsorship networks. A reluctance to engage in casual small talk may also limit relationship building with established leaders, reinforcing structural exclusion.^16^ These dynamics highlight the need for culturally attuned institutional strategies to prevent talented faculty members from being overlooked due to informal gatekeeping practices.

Immigrant faculty members often face heightened challenges in academic medicine, particularly during the early stages of their careers. Many are recruited into roles with near-total clinical responsibilities, limiting opportunities to develop teaching portfolios or pursue leadership development. Visa-related constraints—such as those tied to H-1B, J-1, or green card pathways—often limit faculty from securing federal grants or necessitate full-time clinical work, compromising scholarly productivity and protected academic time.^21,29^ Hiring practices are often shaped by immigration constraints and institutional priorities, including staffing high-demand clinical services and underserved settings.^22^ Compounding these structural limitations, cultural expectations and immigration-related dependencies may further discourage self-advocacy, as faculty members may feel pressure to express silent gratitude rather than assert career needs.^24^

Asian faculty members—particularly immigrants—often experience a lack of social and familial support networks due to geographic separation from extended family and the demands of cultural adaptation.^26,29^ Many relocate independently or with nuclear families, limiting access to traditional support systems typically relied on for childcare, eldercare, and emotional well-being.^26,30^

### Institutional Strategies for Effective Sponsorship of Asian Faculty

Institutional responses to the complex barriers faced by Asian faculty members must be multifaceted and grounded in intersectionality, recognizing the diversity of lived experiences within this group.^8^ Approaches that treat Asian individuals as a monolithic category risk overlooking the compounding effects of structural exclusion, cultural expectations, and identity-based bias. Addressing these issues requires data-informed policies, tailored sponsorship, and explicit recognition of varied advancement pathways. Institutional leaders must be trained to recognize and mitigate implicit biases, particularly those that penalize humility and cultural deference, which can lead to the underrecognition of high-potential faculty members.^8,26,29^ Sponsorship training programs that promote cross-racial and cross-ethnic mentorship have demonstrated success in broadening advancement pipelines.^20^

In addition, creating diverse oversight committees and holding institutions accountable for sponsorship outcomes can prevent unilateral decision-making. Centralized databases of sponsorship opportunities and transparent eligibility criteria may further promote equity. These efforts should be complemented by the creation of affinity groups that represent Asian faculty members and collaborate closely with institutional leadership to identify, strengthen, and sustain equitable sponsorship pathways. These strategies align with broader evidence supporting formalized sponsorship programs, clear nomination protocols, and institutional accountability as critical tools to dismantle persistent advancement barriers for underrepresented faculty.^15,16,20^ When embedded in core organizational policies and combined with culturally responsive practices, these interventions can cultivate inclusive environments that meaningfully support the advancement of Asian faculty members and can be replicated across academic centers nationwide.

### Relationship-Focused Sponsorship for Asian Faculty

This study suggests that traditional definitions of sponsorship—often characterized by a transactional dynamic in which senior leaders advocate for protégés who, in turn, contribute to the sponsor’s professional or institutional goals—may not fully align with how Asian faculty members conceptualize effective professional advocacy.^16,23^ Instead, participants emphasized a preference for relationship-based sponsorship characterized by trust, mutual respect, and interpersonal connection. These relational values reflect broader cultural principles, such as collectivism and respect for hierarchy, that shape how sponsorship is both experienced and valued.^26^ Sponsors who demonstrate authentic understanding of faculty members’ lived experiences were perceived as more effective. These findings underscore the importance of culturally responsive sponsorship models that align with relational norms while maintaining clear goals and institutional accountability.

### Limitations

This study has some limitations. This study was conducted at a single institution, which may limit the generalizability of the findings to other contexts. The participant pool, although diverse in subethnicity, may not fully represent the heterogeneous experiences of Asian faculty members from all geographic regions. Additional research across multiple institutions and using longitudinal designs would enhance the robustness of these insights.

## Conclusions

Asian faculty members encounter distinct cultural and structural barriers to career advancement that are often inadequately addressed in existing frameworks. This qualitative study highlights the need for sponsorship programs that explicitly acknowledge cultural norms, mitigate implicit bias, and incorporate intersectional considerations. Institutional strategies—such as transparent nomination processes, embedding sponsorship metrics into tenure and promotion systems accountability mechanisms, and partnerships with Asian faculty associations—can provide critical oversight. To dismantle the bamboo ceiling, structural reforms must be aligned with cultural awareness, particularly around values such as modesty and deference to authority. A data-driven, culturally responsive approach holds promise for expanding leadership pathways and ensuring that Asian faculty members are fully included in opportunities for advancement. Future research should examine the long-term impact of these interventions on the career trajectories of Asian faculty members in academic medicine.

## Notes

Supplement.
Data Sharing Statement
