# Supplementary material for: Sponsorship and Career Advancement for Asian Medical Faculty
Source: JAMA Netw Open. 2026 Jan 9;9(1):e2553241. doi: 10.1001/jamanetworkopen.2025.53241 (PMC12789948; doi:10.1001/jamanetworkopen.2025.53241)
Supplement: Supplement. — Data Sharing Statement [file jamanetwopen-e2553241-s001.pdf]

Data Sharing Statement

Sebastian. Sponsorship and Career Advancement for Asian Medical Faculty. *JAMA Netw Open*. Published January 09, 2026. doi:10.1001/jamanetworkopen.2025.53241

Data
Data available: No

Additional Information
Explanation for why data not available: Qualitative data from focus groups
